# Naxos disease: from the origin to today

**DOI:** 10.1186/s13023-018-0814-6

**Published:** 2018-05-10

**Authors:** Guo-Liang Li, Ardan M. Saguner, Guy H. Fontaine

**Affiliations:** 1 Department of Cardiovascular Medicine, First Affiliated Hospital of Xi’an Jiaotong University, No. 277 Yanta West Road, Xi’an, Shaanxi 710061 People’s Republic of China; 2 Institut de Cardiologie, Unité de Rythmologie, Hôpital Universitaire La Pitié-Salpêtrière, 47-83 boulevard de l’Hôpital, 75651 Paris, France; 3 Department of Cardiology, University Heart Center Zurich, Rämistrasse 100, 8091 Zurich, Switzerland

**Keywords:** Naxos disease, Sudden death, Plakoglobin, Arrhythmogenic right ventricular dysplasia

## Abstract

Naxos disease, first described by Dr. Nikos Protonotarios and colleagues on the island of Naxos, Greece, is a special form of arrhythmogenic right ventricular dysplasia (ARVD). It is an inherited condition with a recessive form of transmission and a familial penetrance of 90%. It is associated with thickening of the skin of the hands and sole, and a propensity to woolly hair. The cardiac anomalies characterized by ventricular arrhythmias with ventricular extrasystoles and tachycardia and histologic features of the myocardium are consistent with ARVD, but in a more severe form of dysplasia with major dilatation of the right ventricle. The identification of the responsible first gene on chromosome 17, and its product plakoglobin as the responsible protein for Naxos disease proved to be a milestone in the study of ARVD, which opened a new field of research. Thanks to those with the determination to discover Naxos disease, there is and will be more clarity in understanding the mechanisms of juvenile sudden death in the young who have an apparently otherwise normal heart.

## Background

**Fig. 1:**
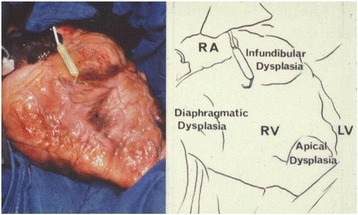
The heart of a 50-year-old female ARVD patient during surgery. The most prominent areas of dysplasia are illustrated on the drawing. Triangle of dysplasia: the most frequent sites of dysplasia: (1) the anterior infundibulum, (2) the right ventricular apex and (3) the inferior or diaphragmatic aspect of the right ventricle (RV). These constitute the original “triangle of dysplasia”. LV - left ventricle, RA - right atrium. (With permission from Marcus FI & Fontaine GH, et al. [7])

**Fig. 2:**
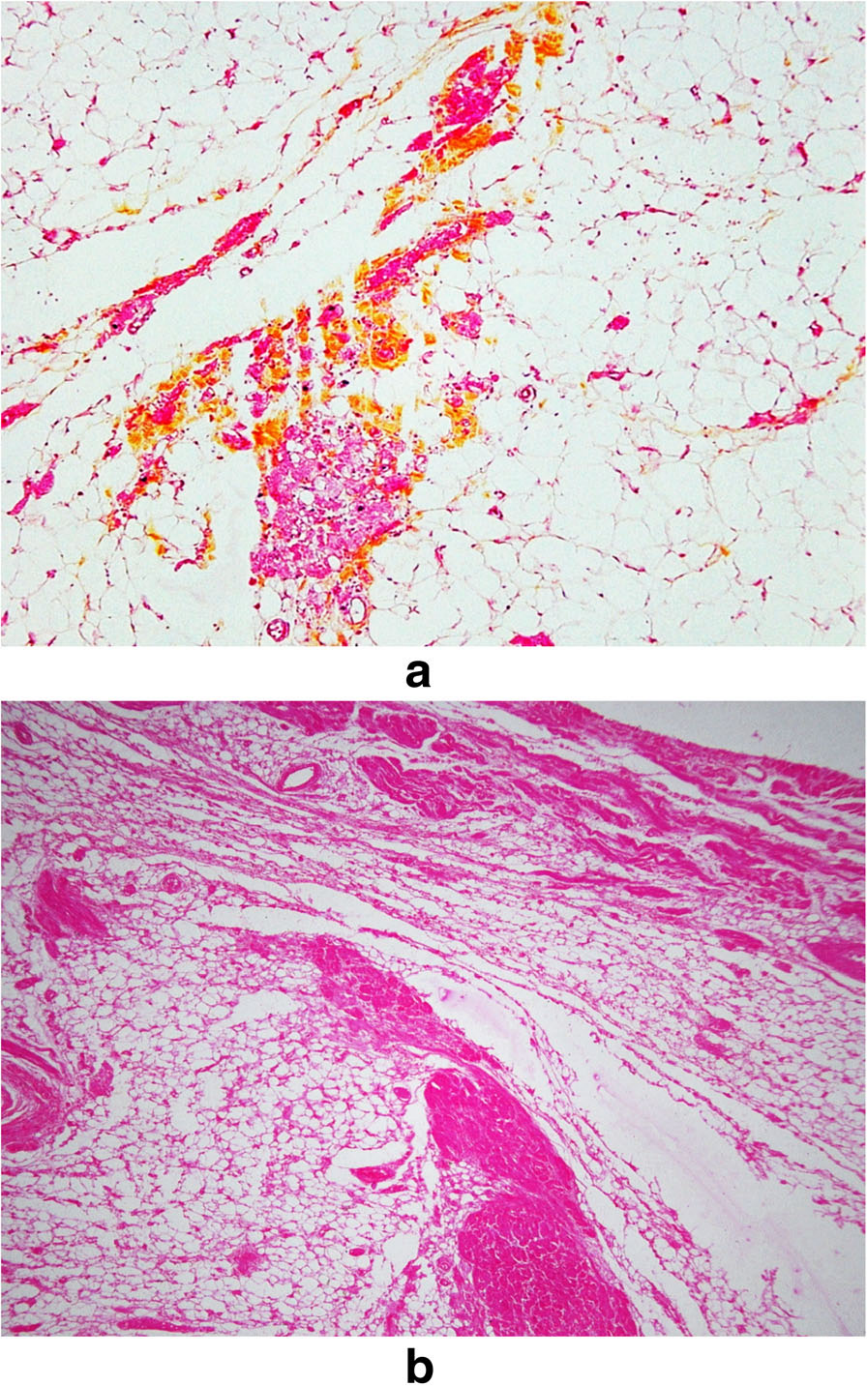
**a**, Surviving strands of cardiomyocytes bordered by or embedded in extensive adipose tissue (magnification× 100). **b**, Most of the myocardial fibers are dissociated by fat and minor fibrosis. (Spongy structure) The pictures were obtained from GF´s personal collection

**Fig. 3:**
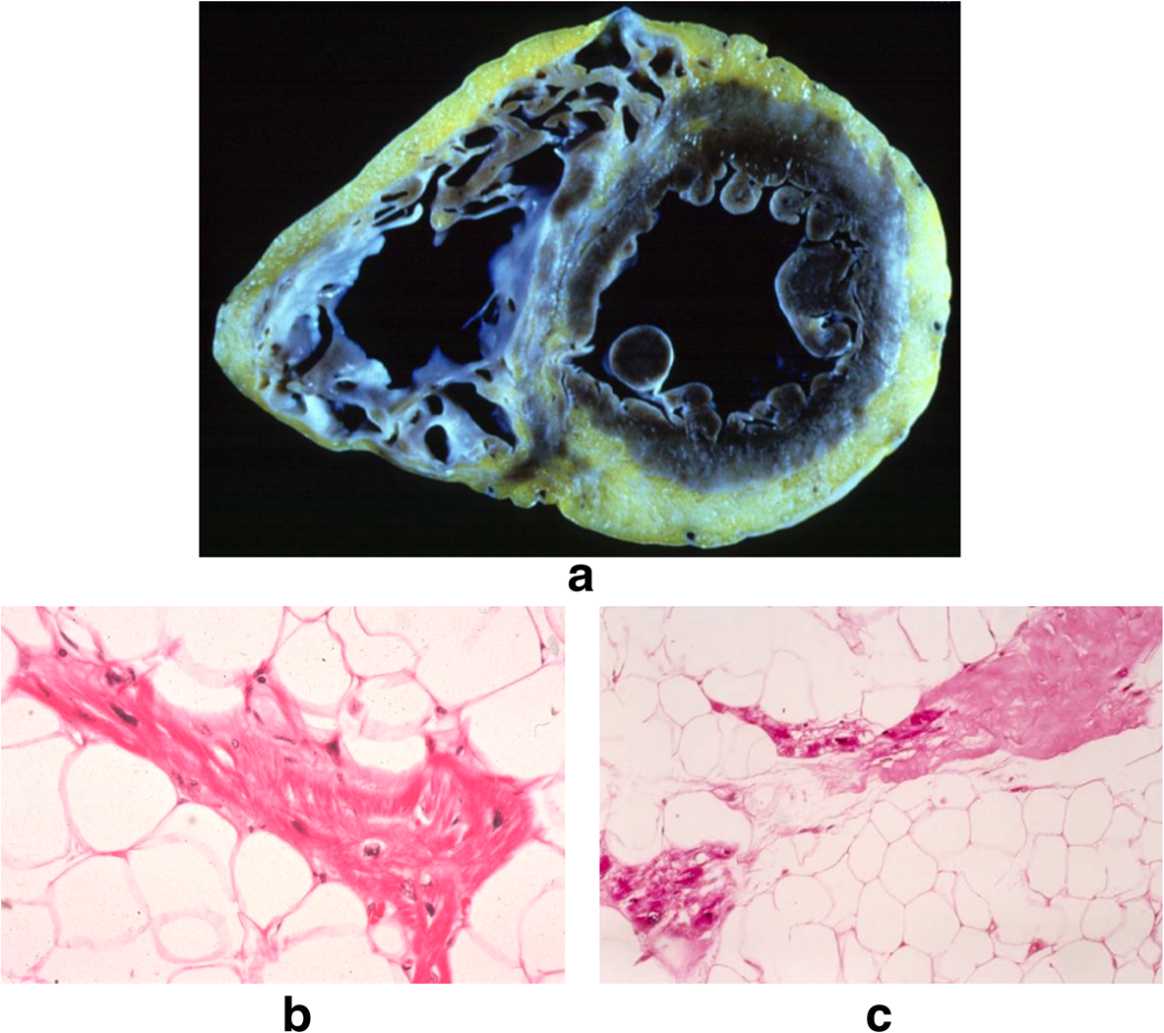
Representative biventricular dysplasia in ARVD. The same disease process, replacement of myocardium by fat and fibrosis, is observed in this patient on the right as well as external part of the left ventricle (**a**). Inside fat, there are surviving cardiomyocytes (**b**) and zones of fibrosis (**c**). (Reprinted with permission from Guy Fontaine, et al. [10])

Arrhythmogenic right ventricular dysplasia (ARVD) was first recognized in 1977 during antiarrhythmic surgery in Pitié Salpêtrière Hospital, Paris, France [1]. The dysplasia predominantly involved the original “triangle of dysplasia” (Fig. 1). The diagnosis of ARVD was pathologically based on previous findings of myocardium embedded in or bordered by fatty tissue and/or fibrosis (Fig. 2) [1–4]. Biventricular involvement (Fig. 3) is very frequently observed at later stages, leading to congestive heart failure and death [5–10]. Naxos disease is a special form of ARVD, which was first described by Dr. Nikos Protonotarios and colleagues on the island of Naxos, Greece [11] and then in other regions [12–21]. It is an inherited condition with a recessive form of transmission and a familial penetrance of 90% [11, 22–27]. It is associated with thickening of the skin of the hands and sole, and a propensity to woolly hair. The cardiac anomalies characterized by ventricular arrhythmias with ventricular extrasystoles and tachycardia and histologic features of the myocardium are consistent with ARVD, but in a more severe form of dysplasia with major dilatation of the right ventricle [23, 25, 26, 28–30]. The identification of the responsible first gene on chromosome 17, and its product plakoglobin as the responsible protein for Naxos disease proved to be a milestone in the study of ARVD, which opened a new field of research [28, 31]. In this review, we provide an impressive historical overview of events and developments that lead to the appearance of the Naxos disease concept in the context of ARVD. The review provides a first-hand account and bears a great deal of personal touch in description of the important historical events.

### The Hellenic era (400 BC)

The most famous of the Greek myths, even though they seem to be deeply rooted in the imagination and springing from the world of legends rather than historical truth, have nevertheless always had the reputation of expressing an aspect of reality. One can ask oneself whether the myth about a famous Marathon runner, who brought the Athenian general Miltiades the victory over the Persians conducted by the king Darius the Great, was the first recorded case of sudden death from ARVD. This story tells of a fit and healthy young man proclaiming, “Victory!” upon his completion of a prolonged and intense physical effort. The youth then collapsed and died suddenly. A closer reading of the texts, which provide accounts of this runner, however, reveal differing views of what actually occurred. Heratus recounts a unique and remarkable version of this story. He describes the messenger Philippides striding to request help from the Spartans, the traditional enemies of the Athenians’, before the dreadful Battle of Marathon. Aristophanes, the first Greek comic poet, transforms the name Philippides into Pheidippides. This name then appears to have the etymology of a man belonging to “a family known for taking care of horses.” Being the best runner of his family, he would have preferred to run the exhausting distance himself in order to spare his steed.

An important step towards learning the genetic aspects of ARVD began with the study of an uncommon illness, which may well have affected lives long ago. This illustrates William Harvey’s well-known saying that the study of uncommon illnesses often allows us to better understand the mechanisms of common diseases. This is indeed what happened, when Naxos disease made its appearance on the medical stage.

The following brief reminder of what the name “Naxos” can evoke in the memories of those, who have been privileged to visit the Greek world, seems an appropriate introduction to describe how Naxos disease was identified.

### Island of Naxos

**Fig. 4:**
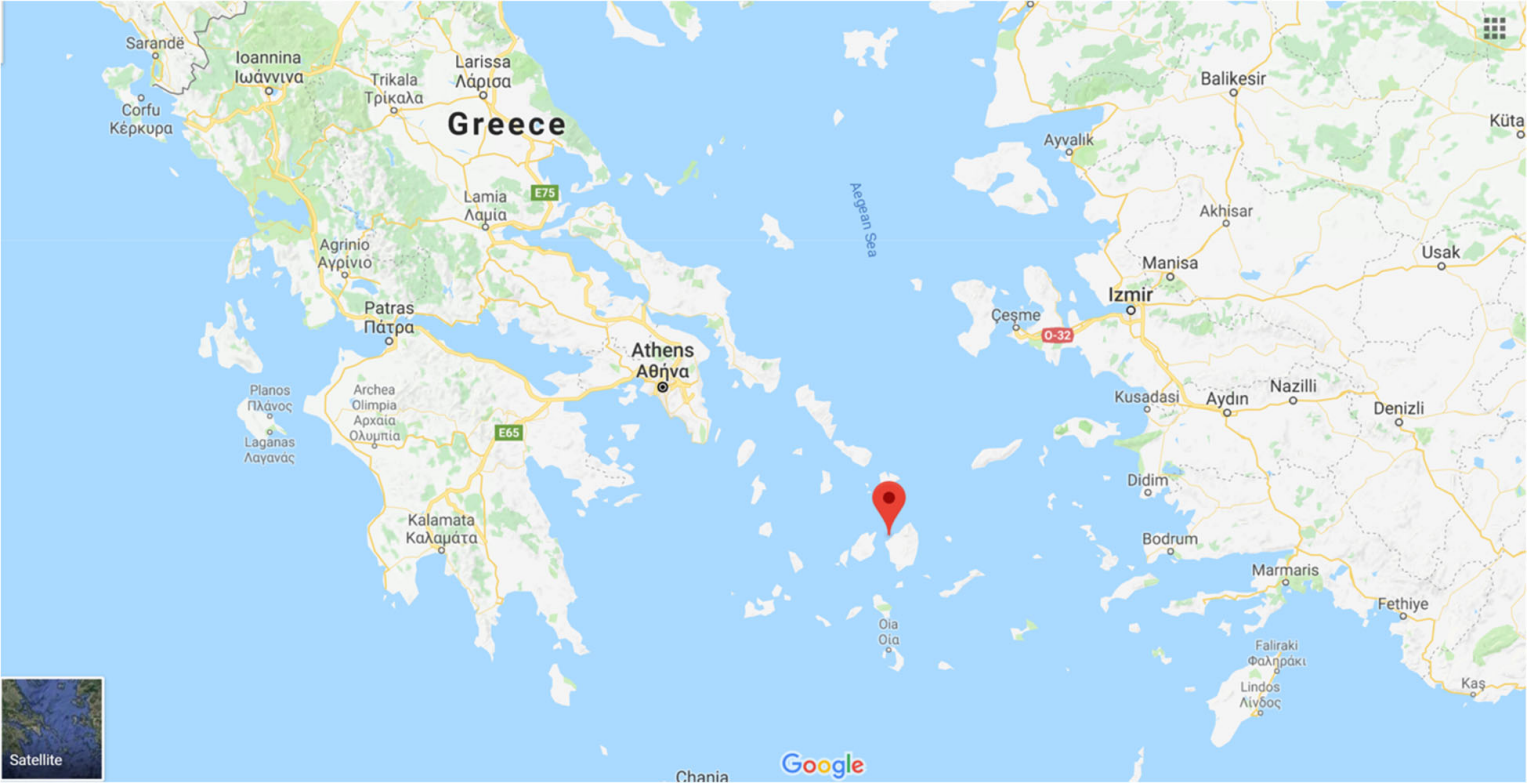
Island of Naxos is situated to the North East of a group of islands called the Cyclades. The original map was obtained from Google Maps

Situated to the North East of a group of islands called the Cyclades, Naxos is the largest one of these islands (Fig. 4). Mountainous and bisected by deep valleys, groups of inhabitants live on the valley floors quite isolated from one another, though less than they were in former times. According to the legend, Naxos was the Isle of Dionysos, the God of vines and wine who was born from the thigh of his father Jupiter. The story blends the power of the gods with their hidden weaknesses. According to another Greek legend, the Island of Naxos was where Ariadne was abandoned by Theseus on his way back to Athens. Theseus had been able to slay the Minotaur thanks to Ariadne who had given him the thread that led him out of the labyrinth. The story, however, says that Theseus boarded his ship and sailed away while Ariadne slept. This melancholic tale has a trace in the French literature, in the beautiful words of Racine’s tragedy “Phedre”:

*“Ariadne, my sister, of what unrequited love did you die on the coast where you were abandoned?”*

Music-lovers may recall the symphonic poem by Albert Roussel or the masterpiece opera by Richard Strauss “Ariadne on Naxos”.

### Athens European cardiology congress (1968)

Quite apart from its cultural resonance, Greece has a very special significance, since it was the country where one of the authors (GF) first achieved international recognition for some of his first work on cardiac pacing. GF had submitted two abstracts to the Athens European Cardiology Congress in 1968. These documents concerned the value of threshold measurements in cardiac pacing, and both were accepted for oral presentation.

### Naxos disease

**Fig. 5:**
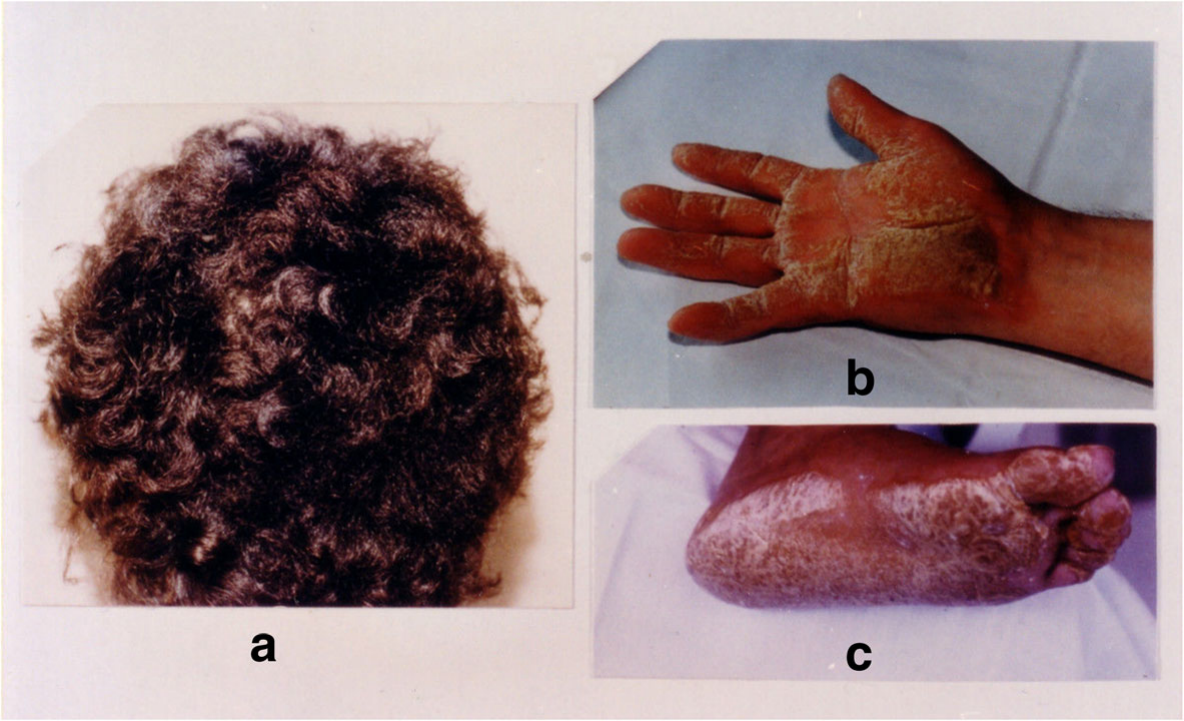
Cutaneous phenotype of Naxos disease: woolly hair (**a**), palmar (**b**) and plantar (**c**) keratosis. Protonotarios N, Tsatsopoulou A. Naxos disease: cardiocutaneous syndrome due to cell adhesion defect. Original materials from an open access article [23]

**Fig. 6:**
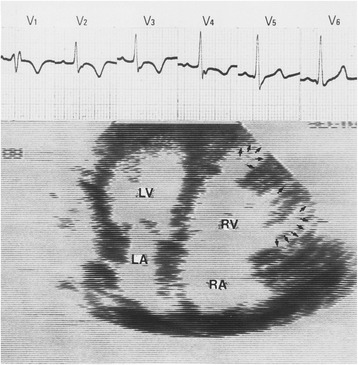
The cardiac abnormality of Naxos disease was not clinically manifest until the patient was ≈15 years of age, and onset was usually with palpitations or syncope. Two-dimensional echocardiogram of the heart showing typical features of ARVD: severe right ventricular (RV) dilatation and multiple saccular aneurysmal segments (arrows). There is also right atrial (RA) dilatation. In contrast, the left ventricle (LV) and left atrium (LA) are normal. Accompanying ECG shows abnormal T-wave inversion in leads V1 through V4, consistent with abnormal repolarization affecting the RV. Epsilon waves are also observed. Original materials from an open access article [23]

**Fig. 7:**
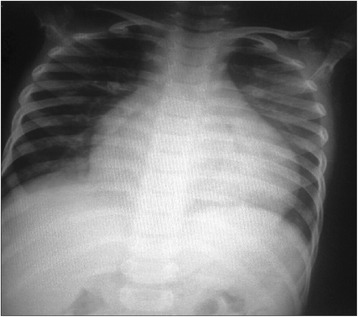
A 6-year-old male child with Carvajal syndrome (variant of Naxos disease): Chest X-ray posteroanterior view showing left-sided cardiomegaly. Original materials from an open access article: [57]

**Fig. 8:**
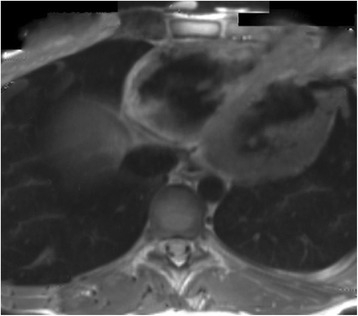
A case of a 14 year old boy from Spain, who was admitted into emergency room after being resuscitated from cardiac arrest, secondary to malignant ventricular tachycardia that developed while he was playing basketball. Four months later he was brought up again to the emergency room after collapsing during mild exercise. His MRI revealed more structural and functional anomalies of the right ventricle, with more myocardial loss and more involvement at layers of posterior and apical walls. The left ventricle was slightly affected. Original materials from an open access article [58]

**Fig. 9:**
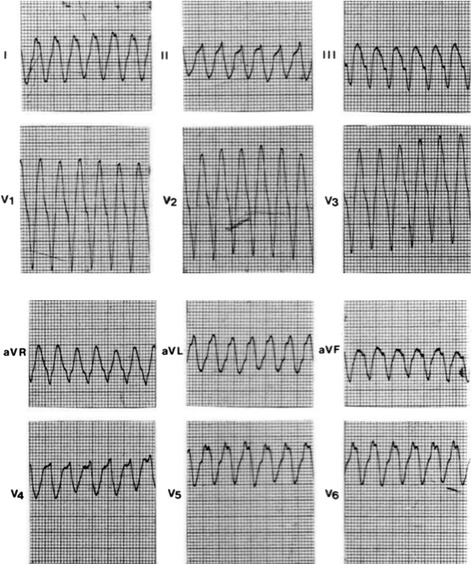
Spontaneous sustained ventricular tachycardia originating from the right ventricular inferior wall, showing a left bundle branch block configuration and superior axis. Original materials from an open access article [23]

**Fig. 10:**
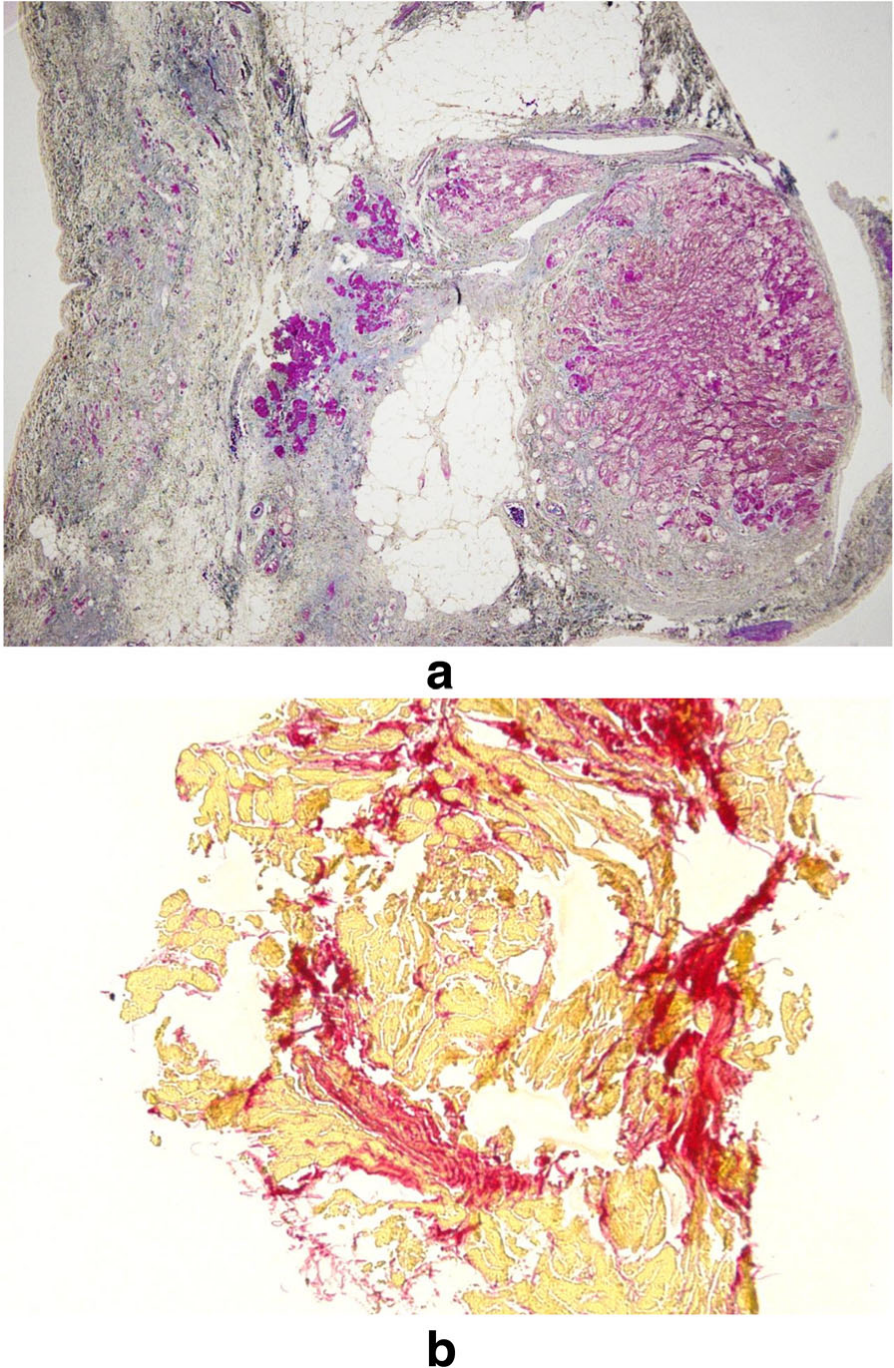
Masson trichrome (**a**, magnification× 40) and Elastica–van Gieson (**b**, magnification× 100) stained section from the right ventricle of a patient with Naxos disease shows surviving strands of cardiomyocytes bordered by or embedded in extensive fibro-fatty tissue.(magnification× 40). (Courtesy of Dr. Nikos Protonotarios, Naxos, Greece)

Indeed it was on this island, rich in reminders of mythological happenings, that a modern-day rural doctor, general practitioner, at that time, made a keen observation. The doctor noted that some of his patients possessed two pathologies apparently independent of one another, and yet unquestionably linked. On the one hand, an ectodermic dysplasia manifested itself in the form of palmoplantar keratosis, i.e. a thickening of the skin of the hands and sole, and a propensity to woolly hair (Fig. 5) [11]. On the other hand, cardiac anomalies were present (Figs. 6, 7 and 8). These were characterized by ventricular rhythm disorders with ventricular extrasystoles and tachycardia (Fig. 9). There were also structural abnormalities with large areas of fibro-fatty tissue bordering surviving fibers of myocardium (Fig. 10).

After having collected various cases, the cardiologist realized that the illness seemed to run in families. He wanted to find the origin of these two such disparate sets of symptoms. The first, the keratosis, was known to Greek dermatologists. It was thought that it could be related to Meleda disease, [32, 33] present in this part of the world and throughout the Mediterranean area, reaching as far as the Dalmatian coast. The cardiac anomaly was more mysterious.

Preliminarily, Dr. Nikos Protonotarios sought the assistance of Greek academics in Athens. The first question to be answered was whether a clinical entity of this sort of disease had ever been previously described. Having learned that it had not, Dr. Adalena Tsatsopoulou decided to consult the most comprehensive library to which they could gain access. The Elsevier Library in Amsterdam offered learned works, journals and books over which the doctors poured for more than a month to search the whole of Index Medicus back to 1895. They finally lit upon an article which triggered their interest. It had been written by a Frenchman in English and was published in a Japanese journal distributed by a German publisher [34]. The article had been written by one of us (GF) after the occasion of the third Mikamo lecture that GF had given in Osaka in 1983. The presentation in Osaka was based on the beginnings of surgery as a cure for ventricular tachycardia (VT), a rather aggressive procedure, which could be replaced by something simpler. The piece in the journal had seemed incumbent upon GF to write something which would draw together the strands of knowledge about ARVD at that time. It was this article, describing a new pathology of the “right ventricle,” that had caught the attention of Dr. Nikos Protonotarios and colleagues. The doctors had a reason to believe that they had identified a new clinical entity whose closest expression in cardiological terms was that of ARVD, common to a number of patients within whom Dr. Nikos Protonotarios observed that both the ectodermic dysplasia and cardiac anomalies were the intractable arrhythmias and cardiac abnormalities in terms of major dilatation of the right ventricle. A groundbreaking paper describing these cases was written by Dr. Nikos Protonotarios and Dr. Adalena Tsatsopoulou and several of their colleagues. In 1986, the *British Heart Journal* published this excellent paper [11]. A unique note in the chart of each patient, who was mentioned in the document, was that they had descended from families on the Greek island of Naxos [11].

### Two Naxians in Paris

Our team in Paris had become particularly skilled in the use of ablative methods by 1987 when, for that reason, we were chosen to receive a Naxian patient. When the 34-year-old patient arrived in the Jean Rostand Hospital, Sèvres, France, the main consideration was his most obvious symptom, that of repeated and intractable VT. A thorough cardiac examination revealed a left ventricle with normal function and all other aspects to be normal. The patient was treated by means of fulguration in the right ventricle after undergoing the usual evaluation procedures in cases of this disorder. Antiarrhythmic therapy, based on programmed stimulation, was also found to be effective. This treatment greatly reduced the number of attacks, which led to the declaration of a satisfactory clinical condition within a few weeks.

A few years later, the second Naxian case was sent. This time, the patient had a healthy condition which seemed to be much more worrying. His left ventricle was affected by major segmentary anomalies of contraction. Angiography results showed a left ventricular ejection fraction of 35%. This patient was also successfully treated.

Looking back over our case notes, we found that we could not file the Naxian cases along with our local dysplasia cases. The first Greek patient presented with palmoplantar keratosis, this did not exist in any of our other cases. The second Greek patient presented with evidence of changes to the left ventricle. This led us to suspect a much deeper myocardial pathology that could not really be classified as a typical right ventricular dysplasia.

### Toward genetics

Some months after treating the second patient, as GF prepared to send his case notes back to Naxos, Greece, GF was struck by the idea that dysplasia could be considered as a “morbid association” of Meleda’s disease. This thought made the second case even more curious, as his angiographies had shown that he was unquestionably suffering from a major left ventricular lesion. These events unfolded at a time when the left ventricle’s involvement in dysplasia had not been entirely elucidated.

Thoughts about this “morbid association” engendered a pressing desire. The histories of the Greek families whose members were affected by these two differing pathologies would need to be explored in greater depth. In order to do this, we would have to contact Dr. Nikos Protonotarios. A letter sent to the Athens’ address mentioned in the *British Heart Journal* [11] article was returned, “unknown at this address”. Fortunately, GF had a very grateful patient living in Athens at the time. Thanks to his assistance, GF was able to track down Dr. Nikos Protonotarios in less than 48 h. Dr. Nikos Protonotarios had returned to his birthplace and established a private clinic. He was a prestigious and practicing cardiologist on the island of Naxos, Greece.

Contacting Dr. Nikos Protonotarios was immediately gratifying. He was willing to collaborate with our team in their efforts to study this strange affliction. We expressed our conviction, to which importance should be attached, was in observing the clinical condition in the Hellenic families, i.e. the association of probable ARVD with visible palmoplantar keratosis. Preliminary studies of the patients’ pedigrees suggested a direction for additional observations. It seemed possible that the condition was transmitted to the subjects inhabiting the island through a recessive gene as stressed in the original publication in the *British Heart Journal* [11].

The situation on Naxos seemed to echo the genetic findings of a study in England [35]. Incidentally, that study was made when myocardial pathologies were just beginning to be identified and were still referred to as cardiomegalia, or simply “enlarged heart”. There were villagers who had intermarried with other inhabitants of their own village. This gave their children a propensity to develop what was evidently hereditary cardiomegalia. Those people who married to the individuals outside of their own villages would have children exempt from the disease. Although there was no evidence of interbreeding, the findings did suggest that endogamous phenomena with possible genetic reinforcement were at work.

Dr. Nikos Protonotarios finally obtained informed consent after long discussions with the affected families on Naxos and collected 12-lead ECGs of the cases he had. The ECGs were brought to the Jean Rostand hospital, Ivry-sur-Seine, France, for further analysis. It appeared that the ECG signatures of the disease found on Naxos were statistically comparable and seemed to be, if anything, more pronounced than those from ARVD. In May 1994, GF visited Dr. Nikos Protonotarios and Dr. Adalena Tsatsopoulou in Naxos. In the space of 3 days GF saw 7 patients, all of whom GF gave thorough clinical examinations. Each case was reviewed by ECG recorded on a computerized Mac-PC Marquette electrocardiograph (American International Medical, California, USA) and echocardiography (Toshiba Machine Co., Ltd., Shizuoka Prefecture, Japan). Each examination was either preceded or followed by a handshake that gave tangible proof of a thickening skin on the palm of the patient’s hand. Individual cases were then discussed on the basis of the available literature, the projection of slides which showed histological images, and texts that could help in understanding the concepts of ARVD. After many interesting discussions and ideas followed, it was convinced that there was a proof of a clearly defined clinical entity in which the signs of palmoplantar keratosis greatly facilitated its diagnosis. These signs were closely associated with right ventricular anomalies on both the structural and the electrocardiographic levels. The term of “Naxos disease” was proposed. GF, Dr. Nikos Protonotarios and colleagues finally presented an abstract that included the name “Naxos disease” at the AHA in 1994 in Dallas, Texas, USA [36].

“Naxos disease” then came into being as a genetically determined family disease. The affliction presented with palmoplantar keratosis, woolly hair, and the typical signs of ARVD, but in a more severe form [13, 23, 27, 29, 30]. The severity corresponded to a more marked phenotypic expression of a genetic mutation such as that governing ARVD. Histological documentation was also in existence. Two patients with Naxos disease had been operated upon for VT in London. Pre-op biopsies on the whole of the right ventricular wall clearly illustrated two aspects that were typical of dysplasia. On the one hand, pure dysplasia was evident. On the other, dysplasia associated with elements that were suggestive of myocardial phenomena involving the left ventricle was observed. The latter phenomena were accompanied by changes in cardiac function.

Dr. McKenna and his team pressed toward the identification of the gene responsible for Naxos disease. They used the genetic identification method of links that allows the comparison of genomes of family members, some of whom are affected and others are not. In 1996, Dr. McKenna and his team further identified a critical zone on chromosome 17 in position q21 [28]. In 1999, McKenna indicated that researchers were on the verge of identifying the gene responsible for Naxos disease at a private meeting which was held in Berlin. In April 2000, several days before setting off to Athens for a meeting of the Greek Cardiological Society, GF received a phone call from Dr. Adalena Tsatsopoulou and was told that the Naxos disease gene had been clearly identified. It involved the mutation of a protein, plakoglobin, considered to be responsible for both the cardiac anomaly and the palmoplantar keratosis. The work identifying plakoglobin as the responsible protein for Naxos disease proved to be a milestone in the study of Naxos disease and ARVD [31]. After the Naxos gene was identified, the pace at which knowledge accumulated speeded up considerably. A “knockout mouse” which was produced in Berlin [37] showed the gene surfacing through anomalies in the cutaneous tissue, but not through adipocytes in the right ventricle. Research on the gene then carried out at an infundibular idiopathic tachycardia level, showed the positive results.

The protein plakoglobin is known to play a part in cellular adhesion at the desmosomal level in cardiomyocytes [12, 38]. It forms points of mechanical connection between cells, and to which filaments of desmin are attached. The armadillo protein plakoglobin is part of the intermediate filaments. It is also homologous with the keratin filaments found in cutaneous tissue. Identification of the gene responsible for Naxos disease made it possible to comprehend a connection between the heart trouble linked to plakoglobin, the protein of the desmosome plaques to which the intermediate desmin filaments attach themselves, and the rupture of mechanical cutaneous desmosome links to which keratin attaches itself. The rupture of these mechanical links produces a reaction, in this case, a hyperplasia of the keratin layer similar to that to be seen in bullate skin complaints. Disassociating these cells could induce a cellular transdifferentiation phenomenon.

### Sudden death from infundibular tachycardia: Genetic explanation

A sixteen year old girl was at school when she jumped up, held her hand to her chest and cried, “Oh ... my heart!” Suddenly she went pale, lost consciousness and collapses. The girl was slow to recover, the emergency services were called. They attempt to resuscitate her but it was too late, she had suffered irreversible brain damage. Although brain-dead, the young girl survived for more a week. During that time, infundibular ventricular extrasystoles and inconsistent flickers of VT were recorded. The young girl suffered from right ventricular outflow tachycardia originating from the infundibular region.

**Fig. 11:**
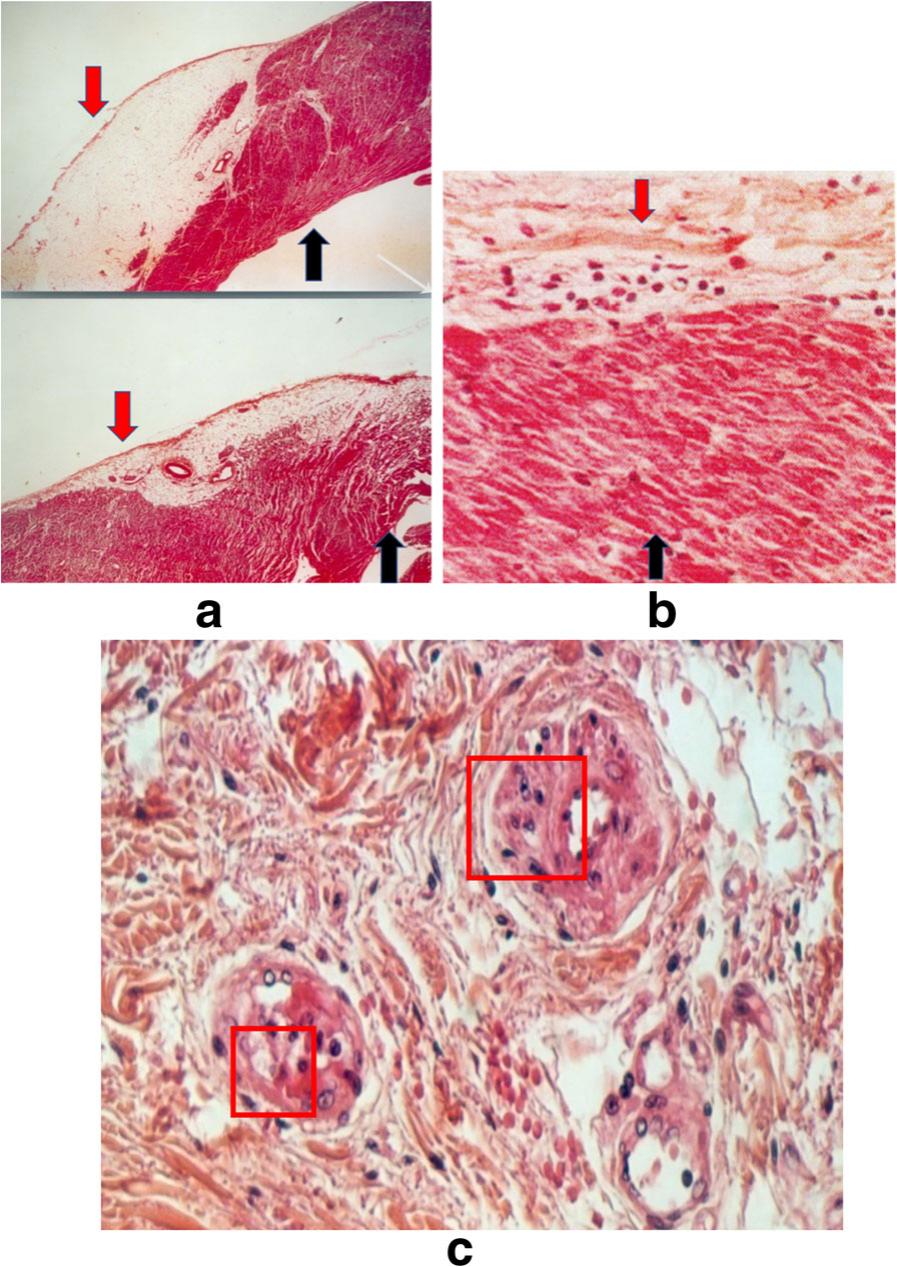
Sudden death in a 16-year-old girl. **a** and **b**, The infundibular area and the adjacent zone with trabeculations show massive adipocytes with surviving strands of cardiomyocytes, suggesting a localized form of ARVD. **c**, Typical pattern of increased thickness of the small coronary vessels. Red arrow, epicardium; Black arrow, endocardium. Red square frame, increased thickness of the media layer. (With permission from Fontaine GH) [10]

The event in 1996 took place in the city of Tours (between France’s Cher and Loire rivers). Informed of it by Prof. Pierre Cosnay, Hospital regional universities Cardiologie, Tours, France, GF requested an autopsy wherein the histological study would concentrate on the infundibular region (Fig. 11). The study not only showed that there were abnormal quantities of adipose tissue in the infundibular region, but that there were also rows of cardiomyocytes going right through this adipose tissue. Fibrosis and inflammation indicative of perimyocarditis, as well as the presence of lymphocytes caught in a particularly well-defined fibrosis, were also found. After reviewing these data, we concluded that the tragedy was caused by a localized form of ARVD with complications arising from myocarditis, whose potential in the acute deterioration of ARVD is known now [39–45]. Considering the absence of other reasonable evidences to be discussed at the time of reviewing the documentation, the indications were that these infundibular ventricular extrasystoles and tachycardia were not as harmless as generally supposed. Similar observations had been recorded in other cases. We know now that a considerable percentage of idiopathic right ventricular outflow tachycardia is localized forms of ARVD.

Several years later, and after the identification of the Naxos disease gene, others who experienced infundibular tachycardia came into focus. The affected patients from the same area as Naxos disease were tested for the plakoglobin gene. All of the patients with Naxos disease were found to be homozygous after genetic testings. The infundibular tachycardia patients represented a heterozygous expression of the illness. Given this information, we formulated a “working hypothesis.” The patients who had suffered from the infundibular tachycardia, in general, were nothing other than the heterozygous expression of the illness. The aforementioned case of the young girl had already highlighted the not entirely harmless nature of infundibular tachycardia. Patients who had experienced these arrhythmias, upon evaluation by means of angiography and magnetic resonance, had shown structural anomalies in the infundibular region.

### Carvajal syndrome

Carvajal syndrome, first described by Carvajal-Huerta in Ecuador in 1998, is a variety of Naxos disease, [13, 16] which has been reported in families from different regions [16–18, 21, 46, 47]. It is usually inherited as an autosomal recessive disorder due to defects in the desmoplakin gene. Its clinical characteristics include woolly hair and palmoplantar keratoderma and others as similar to Naxos disease aforementioned. Carvajal syndrome at the earlier stage during childhood presents with dilated cardiomyopathy (Fig. 7) leading to a left dominant phenotype of ARVD and most of these patients die during adolescence [48]. Evidence for a wide genetic heterogeneity of ARVD, Naxos and Carvajal syndrome were summarised in previous papers [13, 20, 21, 35, 49].

### Recently emerging progress

Science has now identified at least 13 genes responsible for typical cases of ARVD [50]. Therefore, the lessons have been learned through the study of Naxos disease, which first pointed at desmosomal abnormalities. These genes could be tested as candidate genes in patients suffering from infundibular ventricular tachycardia, which is a rather frequent form in the spectrum of ARVC/Ds [51].

Over the past decades, a variety of biotechnologies are emerging and have revolutionized the study of human disorders [52–56]. These advances in biomedicine have led to novel pathogenic insights of ARVD. One of the most exciting crucial roles in revealing the pathogenesis of ARVD is the recapitulation of “ARVD” using patient-specific induced pluripotent stem cells [4, 21, 54]. Of particular interest is the ARVD model published by V. Chen’s group from San Diego, California, USA [54]. The insights of pathological mechanisms of ARVD in this study were consistent with our original hypothesis in 1977 that ARVD is a disorder of cardiac development, [1, 54] renovate the notion that the presence of fatty-fibrosis, not replacement of myocardium by fatty or fibrosis, are the pathological characteristics of ARVD. As we said before, the establishment, evolution, pathogenic findings, and potential therapies of ARVD finally run a full circle, back to where the disease was identified [4]. All of these declarations are also applicable to the study of Naxos disease.

## Conclusion

Thanks to Naxos disease, a pure genetic disorder, a new field of research was opened. Thanks to those with the determination to discover Naxos disease, there is and will be more clarity in understanding the mechanisms of juvenile sudden death in the young who have an apparently otherwise normal heart.

## Abbreviations

ARVC: Arrhythmogenic right ventricular cardiomyopathy
ARVD: Arrhythmogenic right ventricular dysplasia
VT: Ventricular tachycardia
